# Flavor and Metabolite Profiles of Meat, Meat Substitutes, and Traditional Plant-Based High-Protein Food Products Available in Australia

**DOI:** 10.3390/foods10040801

**Published:** 2021-04-08

**Authors:** Kornelia Kaczmarska, Matthew Taylor, Udayasika Piyasiri, Damian Frank

**Affiliations:** 1 CSIRO Agriculture and Food, North Ryde, NSW 2113, Australia; korneliak85@hotmail.com (K.K.); battagodage.p@gmail.com (U.P.); 2 CSIRO Land & Water, Canberra, ACT 2601, Australia; 3 Centre for Advanced Food Enginomics, The University of Sydney, Sydney, NSW 2006, Australia

**Keywords:** plant-based, meat, volatilome, metabolomics, protein

## Abstract

Demand for plant-based proteins and plant-based food products is increasing globally. This trend is driven mainly by global population growth and a consumer shift towards more sustainable and healthier diets. Existing plant-based protein foods and meat mimetics often possess undesirable flavor and sensory properties and there is a need to better understand the formation of desirable meat-like flavors from plant precursors to improve acceptance of novel high-protein plant foods. This study aimed to comprehensively characterize the non-volatile flavor metabolites and the volatiles generated in grilled meat (beef, chicken, and pork) and compare these to commercially available meat substitutes and traditional high-protein plant-based foods (natto, tempeh, and tofu). Solid phase microextraction with gas-chromatography mass-spectrometry was used for elucidation of the flavor volatilome. Untargeted characterization of the non-volatile metabolome was conducted using Orbitrap mass spectrometry and Compound Discoverer^TM^ datamining software. The study revealed greater diversity and higher concentrations of flavor volatiles in plant-based foods in comparison to grilled meat, although the odor activity of specific volatiles was not considered. On average, the total amount of volatiles in plant-based products were higher than in meat. A range of concentrations of free amino acids, dipeptide, tripeptides, tetrapeptides, nucleotides, flavonoids, and other metabolites was identified in meat and plant-based foods.

## 1. Introduction

Increased consumer concern about environmental sustainability, animal welfare, and health impacts of high meat consumption are important factors influencing the increasing demand for traditional high-protein plant-based alternatives and more recent faux-meat or mimetic-meat substitutes [1,2]. With increased demand for alternative non-animal sources of protein, and a consumer-led movement towards flexitarian and more sustainable diets, it is essential to better understand the potential for formation of desirable flavors from plant protein precursors to improve formulations of novel high-protein plant foods, especially those that attempt to replicate meat-like flavor attributes. While the flavor profiles of meats such as beef, chicken, or pork have been extensively characterized in literature [3,4,5,6,7,8,9,10], the flavor of high-protein plant-based products and meat substitutes have not been extensively investigated or reported.

Meats from animal muscles and organs are an excellent source of high-quality complete protein and can also be a source of fats and important micronutrients such as zinc, iron, and vitamin B12 [11]. Meat in its raw form has a relatively mild flavor and needs to be subjected to thermal processing (mainly frying, grilling, and roasting) to promote complex Maillard and Strecker degradation reactions and oxidation of lipids, leading to typical meat flavor and aroma formation [12,13]. Small molecules including free amino acids, peptides, nucleotides, sugars, acids, and thiamine (vitamin B1) are important meat flavor precursors in Maillard, Strecker, and other reactions [14], and also contribute to the development of desirable meaty, savory, umami [15,16], and kokumi [17,18] taste. The intramuscular fat present in meat also plays a critical role in the formation of characteristic volatile aroma compounds and delivery of flavor, as well as the juiciness and mouthfeel of meat [3,19,20]. Overconsumption of processed and red meat is potentially linked to negative health effects, such as cardiovascular disease, diabetes, and cancer [21]. Multiple factors influence the decision to consume meat and probably also other non-animal high-protein foods: an intrinsic human desire for the energy and satiety associated with high-protein and nutrient-rich foods [22]; cultural and social factors; affordability; availability and convenience; umami taste and savory flavor; and other drivers [23,24].

Traditional high-protein soy-based foods like tofu, tempeh, and natto have been widely consumed in Asia for centuries. These foods are produced using microorganisms to preserve, modify, and/or improve the digestibility and sensory acceptance of the final product; however, tofu is normally produced in the West by chemical precipitation of protein rather than the use of microorganisms. Recently, alternative legumes and/or grains (lupin, faba) are also being used in the production of these foods [25,26]. The nutritional and health promoting properties of tofu, tempeh, natto, and other traditional fermented Asian foods are receiving more attention around the world [27]. Apart from being good sources of protein, some of these fermented products may also provide bioactive peptides, vitamins, phytochemicals, dietary fiber, and other metabolites that contribute to the functional benefits of these traditional plant-based foods [27]. The distinctive flavor profiles of fermented foods are mainly due to the enzymatic activity of microorganisms which break down the protein (proteolysis) into smaller peptide fragments and free amino acids as well as breaking down complex carbohydrates into more simple molecules such as sugars [28]. Among the most important flavor-active compounds in hydrolyzed foods, free amino acids, nucleotides, peptides, and their derivatives provide umami and kokumi molecules and impart associated desirable sensory properties [18,29,30]. Tofu, tempeh, and natto are nutritious sources of high-quality protein and are often used as alternatives to meat or fish [31]. Natto has very distinctive sensory properties; the flavor is described as soy sauce, sour, nutty, and chocolate-like and the soybeans develop a unique sticky mucilaginous surface texture produced by *Bacillus subtilis natto* fermentation [32,33]. Raw tempeh has a pleasant slightly fermented odor and after grilling or frying at high temperatures, tempeh may develop desirable “meaty” and “nutty” flavor notes [31]. Tofu is produced through soymilk coagulation using salts, and has a mild and bland flavor which is not meaty at all. When tofu is fried or grilled, Maillard and other high temperature reactions occur on the surface, bringing about only mild savory and nutty flavors [34]. 

“Fake”- or “faux”-meat substitutes are manufactured foods that attempt to replicate the organoleptic (flavor, texture, mouthfeel) and nutritional content (protein, iron, B12 content) of specific types of meat (beef, chicken, or pork) [35]. They utilize non-meat protein sources including mainly texturized soy and other legumes such as pea protein, nuts, cereal, vegetables, and mycoproteins. Texturized soy protein (TSP) is the most common protein component of commercially available plant-based meat alternatives, which are becoming more popular around the world [36]. The soybean protein used to create TSP usually has undergone significant processing to partially purify the protein fraction and remove lipids and to inactivate lipoxygenase activity. Soybeans have a relatively high fat content, and lipoxygenase derived volatiles such as 2-pentylfuran and others are linked to undesirable beany off flavors [37,38]. Good quality TSP generally has a neutral and bland flavor profile and requires the addition of flavor precursors and/or Maillard flavors to create products that taste convincingly meat-like. Globally, the meat substitute market is growing fast, and demand for sustainable, nutritious, and palatable meat substitutes is increasing rapidly [23].

This study aimed to map the essential non-volatile flavor precursors—free amino acids, nucleotides, small peptides, and other small metabolites—and grilled volatile flavor characteristics of commercially available meats (beef, chicken, and pork) and compare them to commercially available meat substitutes and traditional high-protein plant-based foods—natto, tempeh, and tofu available in Australian supermarkets (Sydney).

## 2. Materials and Methods

### 2.1. Chemicals

All chemicals were purchased from Sigma Aldrich (Castle Hill, NSW, Australia) except where stated otherwise. Reference volatiles were used to confirm the identity of the following: Acetic acid, butanoic acid, isovaleric acid, hexanoic acid, ethanol, 1-octen-3-ol, 2-phenylethylalcohol, acetaldehyde, 2-methylbutanal, 3-methylbutanal, (*E,E*)-2,4-hexadienal, ethyl acetate, methyl butanoate, ethyl hexanoate, benzaldehyde, 2,3-pentanedione, 2-pentylfuran, acetone, 2-butanone, 2-heptanone, 2-octanone, 2-hydoxy-3-butanone, trimethyl pyrazine, methyl pyrazine, dimethyl disulfide, carbon disulfide, methional, 2,3-butanedione, 2-ethylfuran, 2-heptanone, dimethyl disulfide, hexanal, 1-butanol, 1-pentanol, methylpyrazine, octanal, 1-octen-3-one, 2,5-dimethylpyrazine, 2,6-dimethylpyrazine, 1-hexanol, dimethyl trisulfide, nonanal, (*E*)-2-nonenal, 3-ethyl-2,5-dimethylpyrazine, 1-octen-3-ol, methional, (*E*)-2-nonenal, (*E,E*)-2,4-decadienal, guaiacol, *p*-cresol, 2-ethylthiophene, and 4-methyl-1-pentanol (internal standard). The following standards were used to confirm the identity of non-volatile compounds: L-aspartic acid, asparagine, L-alanine, creatine, carnosine, carnitine, glutamic acid, glutamine, glycine, L-histidine, L-lysine, L-leucine, L-methionine, L-ornithine, tryptophan, L-tyrosine, L-threonine, D-serine, valine, phenylalanine, L-(+)-proline, and nucleotides and their derivatives cytidine, guanosine monophosphate, guanine, guanosine, hypoxanthine, inosine monophosphate, inosine, uracil, uridine, and xanthine.

Ammonium formate, acetonitrile, methanol, formic acid, and positive and negative ion calibrant solutions (Pierce LTQ Velos ESI) were purchased from Thermo Fisher Scientific (Mulgrave, VIC, Australia).

### 2.2. Materials

A total of 30 products were used in the study. Each product was analyzed in triplicate. Ten commercially available plant-based meat substitutes (MS) were investigated. The meat substitutes were selected based on being described as having typical meat flavor: Five faux-“beef” burgers, two faux-“beef” mince, two faux-“beef” sausages, and one faux-“pork” roast. It should be noted that there are many plant-based products (not examined in this study) that do not attempt to imitate meat flavor at all; for some consumers typical meat flavor and texture is not considered desirable. Four samples of tempeh (T), one made from traditional soy and the others made from alternative legumes; two samples of soft or firm tofu (TO); five samples of natto (N), raw and cooked (NC); three beef (B) samples, including regular mince, premium mince, and steak (scotch fillet); three chicken samples (C), including breast premium thigh; and three pork (P) samples, including sirloin steak, loin steak, and cutlet, were purchased from local supermarkets. Natto (N) was locally obtained from an Asian grocery store. The type of product, main ingredients, and nutritional information (available from the original packaging) are presented in Table 1. 

### 2.3. Sample Preparation and Cooking Protocol

Chilled meat samples were removed from retail packaging and minced into small pieces using a hand-blender, and patties were formed (~20 g) and pan-fried (non-stick coating frypan, Mascot, NSW, Australia) using an induction cooktop set to ~ 200 °C (Electrolux, Mascot, NSW, Australia) for 2 min each side or until an internal temperature of 75 °C was reached, measured with a wire thermocouple (FoodPro Plus, Fluke, Baulkham Hills, NSW, Australia). Tempeh and tofu were fried for 8 or 10 min each side, respectively, or until golden. Natto samples were analyzed either uncooked or fried for 8 min each side or until golden. 

### 2.4. Sensory Analysis

Products were evaluated using a free-choice profiling method by five experienced flavor and sensory scientists (4 female, average age 40) in an informal focus group. The main sensory characteristics and attributes were discussed and recorded in the following order: Aroma, taste, texture, and mouthfeel.

### 2.5. Volatile Analysis

Cooked (or raw) samples were homogenized with water at a ratio of 1:2 and a slurry (3 g) was transferred into headspace glass vials. An internal standard (4-methyl-1-pentanol) was added (0.5 µg/g). 

Headspace analysis of samples was performed using solid-phase microextraction (SPME) and gas chromatography—mass spectrometry (GC-MS, Shimadzu QP-2010 Plus, Tokyo, Japan) and an auto-sampler (AOC-5000, Shimadzu, Rydalmere, NSW, Australia). Divinylbenzene/carboxen/polydimethylsiloxane SPME fibers (23′ gauge, 2 cm, Agilent Technologies, Bellefonte, PA, USA) were used for volatile extraction at 40 °C for 60 min and desorbed in the splitless mode into the GC injector (240 °C for 5 min). 

Compounds were separated on a Zebron-WAX capillary column (length 30 m, ID 0.25 mm, and thickness 0.50 µm, Phenomenex, Lane Cove West, NSW, Australia). The carrier gas was helium (1.04 mL/min flow rate). The initial column temperature was held at 35 °C for 5 min, then increased to 250 °C at 5 °C/min and held for 5 min. Detection of volatiles was performed in electron ionization mode (EI), 70 eV over a mass range *m*/*z* 40–250. Kovats retention indices (RI), EI mass spectral library matches, and, in most cases, reference chemicals (R) were used for identification. Except for natto, volatiles were only measured in the cooked samples as they are all typically eaten after grilling or thermal processing. The volatile data were semi-quantified based on the response against the internal standard (4-methyl-1-pentanol), normalized, and then multiplied by the final concentration of the internal standard, assuming a response factor equal to one for all the compounds and expressed as a concentration (µg/g). 

### 2.6. Extraction of Non-Volatile Metabolites

Uncooked samples were homogenized using a hand blender (600 Watt, Braun, Germany) and mixed with 70% methanol at the ratio of 1:2. Extraction was conducted using a TissueLyser (Qiagen Retsch MM300, Haan, Germany) for 15 min. Samples were then centrifuged (Model 1-15, Sigma Laborzentrifugen, Osterode am Harz, Germany) at 18,000 r.c.f. for 15 min. The supernatant was collected, and the residue re-extracted under the same conditions. The supernatants were mixed and filtered using nylon filters (Phenex, 0.2 µm, Phenomenex, Lane Cove West, NSW, Australia). 

### 2.7. Identification of Metabolites Using Liquid Chromatography—Mass Spectrometry LC-MS

Liquid chromatography analysis was performed using a Dionex chromatograph equipped with a pump, autosampler, column compartment and diode array detector (Ultimate 3000RS, Thermo Fisher Scientific, Scoresby, VIC, Australia). Chromatographic separation of compounds was performed on an Intrada Amino Acid column (length 150 mm × I.D. 3 mm; particle size 3 µm; Imtakt, Portland, OR, USA). The mobile phase consisted of acetonitrile/0.1% formic acid (solvent A) and 100 mM ammonium formate (solvent B). A constant flow rate of 0.6 mL/min was used with a gradient elution program: 14% B (0 min), 100% B (3 min), 100% B (10 min), 14% B (12.5 min), and 14% B (15 min). The injection volume was 5 µL.

Accurate mass measurement of metabolites was conducted on a Q-Exactive^TM^ Orbitrap LC-MS (Thermo Fisher Scientific) equipped with a heated electrospray ionization (H-ESI) source. The source conditions were as follows: Spray voltage (positive ion 3.9 kV), sheath gas 60 (arbitrary units), auxiliary gas 10 (arbitrary units) and sweep gas 1 (arbitrary units), capillary temp 350 °C, and auxiliary gas heating temp 400 °C. Mass spectra were acquired in data-dependent workflow in positive and recorded over the mass range of *m*/*z* 70–500 (Xcalibur^TM^ 4.3, Thermo Fisher Scientific).

### 2.8. Identification of Metabolites Using Compound Discoverer Software

Compound Discoverer Ver 3.1 (Thermo Fisher Scientific) was used for identification of untargeted metabolites using a standard workflow template for food science. ACToR (Aggregated Computational Toxicology Resource, U.S. Environmental Protection Agency, USA); FDA UNII—NLM (U.S. Food and Drug Administration, MD, USA); FooDB (The Food Database, The Metabolomics Innovation Centre, Edmonton, Alberta, Canada); and the Peptides databases were selected for identification of compounds. Only compounds with a high mzCloud match (> 60 with majority > 80) were assigned an identity and used in subsequent analyses. 

### 2.9. Statistical Analysis

Initial data manipulation and analysis was conducted using Microsoft Excel and R version 4.0.1 using the tidyverse package [39]. Normalized and semi-quantified volatile data were subjected to MANOVA analysis using GenStat 19th (VSN-International, Hemel Hempstead, UK) statistical package. 

## 3. Results 

### 3.1. General Description of Main Sensory Attributes of Products

Both raw and cooked natto had similar very strong odors, described as coffee, caramel, meaty, and, for some samples, ammonia-like (Table 2). It should be noted that natto is most often consumed cold or at room temperature [40]. The fried tempeh had fermented cider, beany and meaty aroma qualities, and a sour and umami taste. Fried tofu had beany, baked, and mild nutty flavors and was slightly bitter and quite beany in taste. The meat substitutes had variable flavor characteristics as expected from the differences in their listed ingredients. Many had meaty and herb-like flavors as well as grainy, acidic, and salty characteristics.

### 3.2. Volatile Analysis 

**Figure 1:**
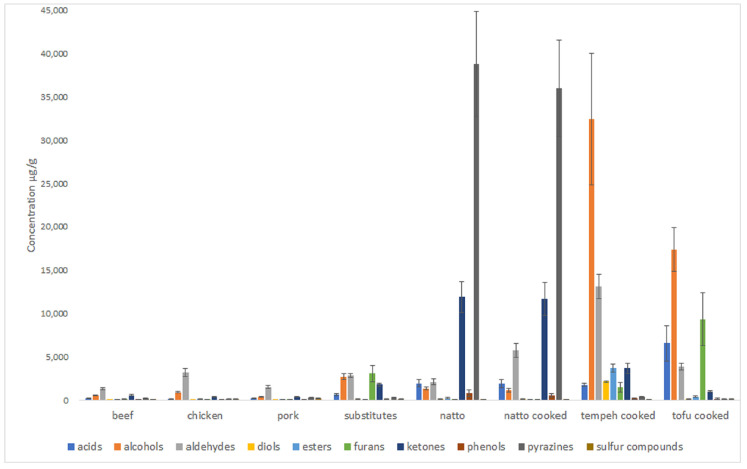
Volatile profile of meat, meat substitutes, natto, tempeh, and tofu according to chemical class. The concentration of each compound is normalized and calculated based on the internal standard (4-methyl-1-pentanol) assuming a response factor equal to one for all compounds.

A total of 98 volatiles were identified across the meat, meat substitutes, and fermented plant food products using the SPME GC-MS method (Table S1). Obvious qualitative and quantitative differences between the volatile profiles of meat and plant-based products were observed (Figure 1). Overall, the traditional plant-based products (natto, tempeh, and tofu) had a higher concentration of total volatiles compared to meat and meat substitute products. The concentration of total volatiles in natto and tempeh was approximately 10 times higher than in the meat samples. The volatile profile of the natto was dominated by alkylpyrazines and ketones, whereas the tempeh was dominated by alcohols and aldehydes. The cooked tofu had a higher concentration of most volatile classes compared to meat, especially alcohols, furans, and organic acids (acetic acids). The concentration of volatiles in the meat substitutes was generally higher than in meat, with a relatively high concentration of alcohols (mainly ethanol), furans (mainly 2-pentylfuran), and ketones, mainly 2,3-butanedione (diacetyl) and 3-hydroxy-2-butanone (acetoin). It should be noted that no consideration of the odor quality or differences in odor activity of volatile compounds was considered in this study. 

Chicken had a higher concentration of aldehydes and alcohols relative to beef and pork. Natto had high concentrations of 2,3-dimethylpyrazine, trimethylpyrazine, tetramethyl pyrazine, 3-ethyl-2-5-dimethylpyrazine, 2,3,5-trimethyl-6-ethylpyrazine, 2,3-butanedione, 3-hydroxy-2-butanone (acetoin), 2,4-(*E,E*)-hexadienal, and isovaleric acid. 

Ethanol, 2-phenylethylalcohol, (*E*)-2-butenal, 3-hydroxy-2-butanone, acetaldehyde, (*E,E*)-2,4-decadienal, and 2-pentyfuran were the most concentrated volatiles in cooked tempeh headspace (Table S1). These compounds were reported previously as important flavor volatiles produced during fermentation of soybean using *Rhizopus oligosporus* [41]. A series of ethyl esters (ethyl acetate, ethyl propionate, ethyl-2-methyl butanoate, ethyl-3-methyl butanoate, ethyl hexanoate, ethyl ethanoate and ethyl octanoate, and methyl benzoate were highly concentrated in tempeh, when compared to other products. Both raw and cooked tempeh are described as having strong fermented flavor attributes. Grilled meat flavor was also described as an attribute (Table 2) in the fried tempeh. Tempeh had a relatively high concentration of total pyrazines, dominated mainly by trimethylpyrazine and 3-ethyl-2,5-dimethylpyrazine. 

Quantitatively, the main volatiles identified in tofu were 1-octen-3-ol, 2-pentylfuran, acetic acid, 1-hexanol, and hexanal. 

Meat substitutes had a higher concentration of total volatiles, when compared to beef, chicken, and pork. Overall, 2-pentylfuran and d-limonene were the most abundant volatiles in the meat substitutes. In other respects, the plant-based products had similar volatile profiles to the meat samples (Table S1).

Chicken had the highest average concentration of total volatiles among meat samples, followed by pork and beef, which is in general agreement with previous studies [42,43]. The fat derived volatiles, hexanal, 1-octen-3-ol, nonanal, and pentanal, were dominant in chicken. A similar flavor profile of chicken was reported by Schindler et al. [42]. Hexanal, benzaldehyde, 1-nonanol, 3-hydroxy-2-butanone, and 2-methylbutanal were the most concentrated volatiles in beef [44], while hexanal, nonanal, octane, carbon disulphide, and 1-octen-3-ol were highest in pork, which is mostly in agreement with previously published data [45].

### 3.3. Non-Volatile Metabolites

**Figure 2:**
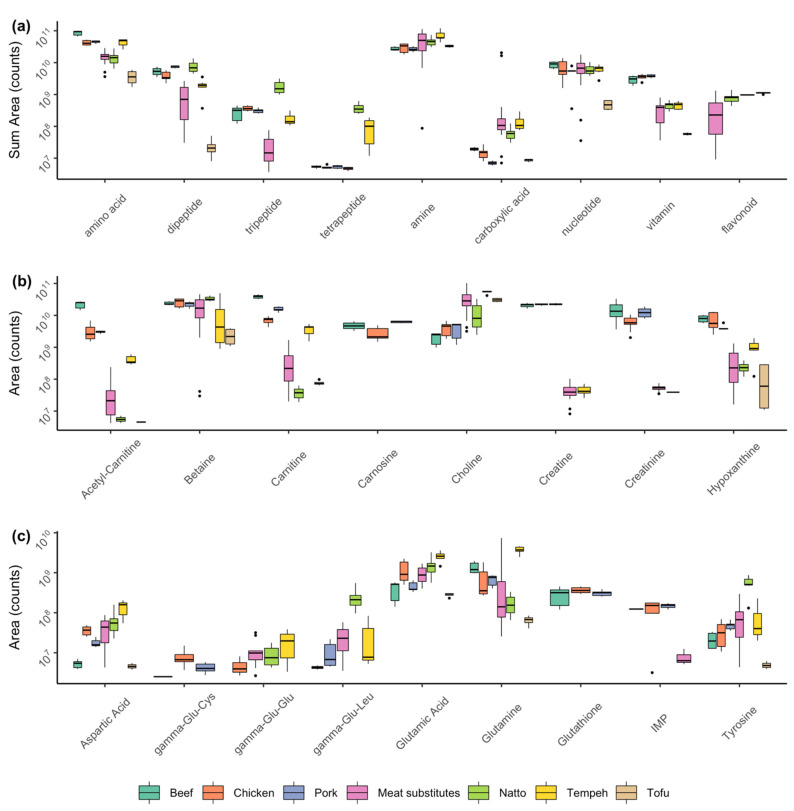
Non-volatile profile of meat, meat substitutes, natto, tempeh, and tofu. Box plots of: (**a**) All compounds from the major compound classes were summed, each sample was classified as either beef (green), chicken (orange), pork (blue), meat substitute (pink), natto (light green), tempeh (yellow), or tofu (beige); (**b**) the dominant amines in each sample group; (**c**) the dominant amino acids, di-, and tri-peptides; IMP is inosine 5′-monophosphate. Box plots show the median, 1st and 3rd quartiles, minimum and maximum values and the dots represent the outliers.

A total of 150 compounds (with an mzCloud match score of > 60) were identified by the Compound Discoverer software: 30 free amino acids, 45 dipeptides, 25 tripeptides, 5 tetrapeptides, 11 nucleotides, 5 flavonoids, 2 pyrazines, and other unclassified compounds were identified in meat and plant-based foods (Table S2). As expected, the muscle meat products were abundant in the nitrogenous compounds L-carnosine, L-carnitine, acetyl-L-carnitine, and creatine (Figure 2a,b). Chicken and beef had the highest concentration of creatine, followed by L-carnitine and acetyl-L-carnitine. Betaine (trimethylglycine), choline, and hypoxanthine were also found in high concentrations in meat. Glutamic acid, aspartic acid, and glutamine (Figure 2c) were also highly abundant in beef, chicken, and pork and these free amino acids are known to directly contribute to the typical taste of meat [3]. When free amino acids (and other non-volatile substrates) on the surface of meat (or meat analogues and other high-protein foods) encounter typical grill temperatures they can participate in Maillard and Strecker reactions to form desirable meaty and grilled flavor volatiles [3,20]. 

The traditional fermented products natto and tempeh were abundant in free amino acids and dipeptides. The free amino acids tyrosine, methionine, leucine, serine and glycine were the most concentrated in natto. Tempeh showed high concentration of glutamic acid and aspartic acid and the highest concentration of γ-aminobutyric acid (GABA). The glutamyl-dipeptides, γ-Glu-Leu, and γ-Glu-Glu were both abundant in natto. Many of the di- and tri-peptides were detected in natto and/or in tempeh including Val-Asp, Pro-Thr, Leu-Leu, Val-Met, Val-Pro, Pro-Met, Gly-Phe, Ala-Tyr, Thr-Tyr, Val-Glu, Gln-Trp, Glu-Pro, Ala-Pro, Gln-Tyr, Val-Asn, Pro-Gln, Gln-His, Gly-Lys, Val-His and Val-Pro-Leu, Phe-Tyr, Thr-Pro, Leu-Gln, L-Arg-L-Ala, Gly-Leu-Pro, Ala-Glu-Leu, and Glu-Ala-Pro. Four γ-glutamyl peptides: γ-Glu-Leu, γ-Glu-Glu, γ-Glu-Cys, and γ-Glu-Cys-Gly (glutathione) were identified in meat and some plant-based products (Figure 2c). γ-Glu-Leu was predominantly found in natto, some meat substitutes, tempeh, and in low concentration in pork cutlets and chicken thighs. γ-Glu-Glu was present in meat substitutes, natto, tempeh, and chicken samples. Glutathione was found in all the beef, chicken, and pork samples, while the glutathione precursor, γ-Glu-Cys, was only identified in chicken and pork samples. 

## 4. Discussion

Undesirable flavors of tofu characterized as beany, grassy, and bitter are associated with oxidation of polyunsaturated (PUFA) lipids by lipoxygenases (LOX) present in soybeans [37,46,47]. Soybeans are known to contain up to 50% of linoleic acid and up to 11% of linolenic acids—both PUFAs are susceptible to oxidation and associated with the formation of undesirable flavors in soy products [47]. The same oxidation of lipids may lead to the formation of the beany flavor of tempeh [41]. 1-octen-3-ol, 2-pentylfuran, 1-hexanol, and hexanal were reported previously as the main volatiles in tofu contributing to green and beany notes in soymilk. These volatiles presented 55% of total volatiles in tofu and are formed during degradation of polyunsaturated fatty acids [36]. Formation of esters have been linked with the growth of yeast and/or fungi in tempeh fermentation, leading to modified sensory properties of the final product [48].

It is well known that volatiles differ widely in their odor-activity value, and the highest odor-impact volatiles typically constitute only a small fraction of the total volatiles and are often present at very low concentration [49]. For example, the odor threshold concentration of alcohols are typically orders of magnitude higher than for aldehydes. 

Pyrazines are important contributors to natto typical flavors and are already present in abundance in the raw natto and are related to strong toast/nut-like odor [32,50]. 2,5-dimethylpyrazine and tetramethylpyrazine are also formed during solid state fermentation of soybean using *Bacillus subtilis* [51] Most of these pyrazines have been reported previously in cooked meat aroma [3,52,53,54], with the notable exception of tetramethylpyrazine. Trimethylpyrazine and 3-ethyl-2,5-dimethylpyrazine are also important contributors to the roast or grilled beef aroma [3,5,55].

The meat substitutes examined in our study were produced from a variety of main protein ingredients (Table 1), some with the addition of spices and other ingredients, leading to increased complexity of the volatile profiles. Notably, higher concentration of 2-pentylfuran in comparison to meat samples may indicate the presence of soy and/or other legumes. The monoterpene d-limonene is present in many herbs and spices and may be considered a marker of the addition of dried herbs [56].

Volatile aldehydes including hexanal, heptanal, octanal, and nonanal are formed during oxidation of fat and are abundant in beef, chicken, and pork, and make an important contribution to cooked meat flavor [3,42,43]. An elevated concentration of aldehydes (particularly hexanal) is often used as an indicator of freshness/flavor deterioration of meat [44,57]. Hexanal, 1-octen-3-ol, nonanal, and pentanal, were dominant in chicken. These volatiles are formed through the thermal oxidation of unsaturated fatty acids such as linoleic acid present in neutral lipid. Chicken fat is relatively high in linoleic acid [58].

Non-volatile analytes are important flavor compounds and flavor precursors, and their impact on the flavor profile depends on cooking conditions. In cooked meat, free amino acids, peptides, sugars, nucleotides, and thiamine are the main flavor precursors [14]. L-carnitine and creatine are considered as bioactive components of meat, playing a role in muscle energy metabolism [59,60]. Betaine has been shown to contribute to the umami flavor of seafoods [61]; choline is an essential nutrient involved in the biosynthesis of membrane lipids [62]. Hypoxanthine is formed from purine degradation and enhances meat taste [63]. Glutamic acid, aspartic acid, and glutamine contribute to meaty and umami flavor characteristics of meat. Both cysteine and glutathione are important precursors for the Maillard reaction products observed in meat flavor. Glutathione and γ-glutamyl peptides are also recognized as kokumi-imparting molecules reported previously in edible beans and matured Gouda cheese [47,64] which may have a role in flavor modification and improving the mouthfeel, thick flavor, and enhancing the intensity of continuity [17]. 

Free amino acids and dipeptides are important flavor precursors in foods. In fermented foods such as tempeh or natto, free amino acids and peptides are main contributors to its characteristic flavor [65,66]. Enzymatic hydrolysis of proteins during fermentation can lead to bitter taste related to the formation of a high concentration of hydrophobic bitter peptides and bitter free amino acids (leucine, valine, isoleucine, arginine, phenylalanine, tyrosine, and tryptophan) [67]. However, some of the peptides formed contribute to desirable umami taste, especially glutamyl dipeptides [28,66]. Kim et al. [40] reported that the fermentation of natto leads to an increase of all free amino acids with the greatest increase of glutamic acids, lysine, tyrosine, and phenylalanine. Increased formation of free amino acids and dipeptides has also been reported to increase meaty and umami flavor of fermented soybean [65]. Serine and glycine contribute to an increased perception of umami flavor of inosine monophosphate (IMP) in soy sauce [68]. The glutamyl-dipeptides, γ-Glu-Leu, and γ-Glu-Glu were shown to impart kokumi sensations [64]. Aspartic acid and glutamic acids are both known to contribute to kokumi and umami flavor character [28,66].

Many di- and tri-peptides have been reported in literature to have bioactive properties. For instance, Val-Asp, Pro-Thr, Leu-Leu, Val-Met, Val-Pro, Pro-Met, Gly-Phe, Ala-Tyr, Thr-Tyr, Val-Glu, Gln-Trp, Glu-Pro, Ala-Pro, Gln-Tyr, Val-Asn, Pro-Gln, Gln-His, Gly-Lys, Val-His, and Val-Pro-Leu present in natto and the majority present in tempeh may act as anti-diabetic agents as they were reported to have dipeptidyl peptidase IV inhibition properties [69,70]. Furthermore, the peptides Phe-Tyr, Thr-Pro, Leu-Gln, L-Arg-L-Ala, Gly-Leu-Pro, Ala-Glu-Leu, and Glu-Ala-Pro, abundant in natto and in tempeh, possess angiotensin-converting enzyme inhibition (ACE) properties and may help in reducing hypertension [69,70]. GABA, found in tempeh, is produced primarily through decarboxylation of glutamic acid and is known to possess antioxygenic and hypotensive effects in rats. It is also an inhibitory neurotransmitter and is used to relieve symptoms such as sleeplessness, depression, and autonomic disorder [27].

The meat substitutes varied in the composition of free amino acids, peptides, and nucleotides compared to meat and traditional plant-based foods. This is likely to be due to the variety of ingredients used to formulate the products. Food manufacturers may add mixtures of free amino acids, nucleotides, and sugars, yeast extracts, protein hydrolysates, and fermented ingredients to create meat-like flavors. Glutamic acid, aspartic acid, monosodium glutamate, and 5′ribonucleotides are often added to non-meat products to enhance savoury, meaty, and umami flavors [28,71].

## 5. Conclusions

The flavor profiles of natto, tempeh, and tofu were very different to the flavor profiles of meat and meat substitutes. While the volatile profile of cooked meat is predominately influenced by aldehydes and alcohols, traditional plant-based foods show a more diverse volatile profile, highly influenced by the fermentation microorganisms and/or other processing method, as in the case of tofu. Natto is a very rich source of potentially bioactive peptides and taste-active compounds when compared to meat. With further processing (heating, drying), natto (and isolated fractions) could be added back into plant-based meat products to increase umami and kokumi flavors as well as improve nutritional profiles.

## Figures and Tables

**Table 1:** Nutrition information of meats, meat substitutes, and traditional plant-based high-protein products.

	Product	Main Protein Source	Protein [g/100 g]	Fat [g/100 g]	Carbohydrate [g/100 g]	Sodium [mg/100 g]	Iron [µg/100 g]	Zinc [µg/100 g]	Vit B12 [µg/100 g]
Meat substitutes									
MS1	Beef Burger	black beans	6.7	4.9	16.2	400	n/a	n/a	n/a
MS2	Beef Burger	vegetables	5.6	7.6	31.5	320	n/a	n/a	n/a
MS3	Beef Burger	brown rice	12.9	6.3	19.2	598	n/a	n/a	n/a
MS4	Beef Burger	soy protein	15.6	0.9	18.6	473	n/a	n/a	n/a
MS5	Beef Burger	pea protein	17.7	17.7	4.4	380	n/a	n/a	n/a
MS6	Beef Mince	mycoprotein	14.9	1.9	1.6	58	n/a	n/a	n/a
MS7	Beef Mince	soy protein	18	10	6.2	480	n/a	n/a	n/a
MS8	Sausage	wheat, gluten and soy	19	10.4	9	480	3.5	4.4	2
MS9	Sausage	soy	8.4	7.4	13.1	630	n/a	n/a	n/a
MS10	Pork roast	wheat, gluten and soy	16.9	5	13.3	590	1.7	1.5	1.7
Plant-based high-protein foods									
T1	Tempeh	soy	20.2	5.9	0.5	3.1	2.4	n/a	n/a
T2	Tempeh	chickpea	12.8	1.9	19	3.2	2.5	1.4	0.04
T3	Tempeh	fava beans	14.7	0.6	15	40	2.1	1.2	0.04
T4	Tempeh	split pea	14.2	1.2	21	3.2	2.7	1.5	0.04
TO1	Tofu	soy	15.2	7.1	1.7	<6	5	n/a	n/a
TO2	Tofu	soy	5.4	1.2	2.1	<1.0	n/a	n/a	n/a
N1	Natto	soy	16.4	10	12	2	n/a	n/a	n/a
N2	Natto	soy	15.2	8	12.9	454	n/a	n/a	n/a
N3	Natto	soy	13.2	7.8	15.1	562	n/a	n/a	n/a
N4	Natto	soy	14.8	18	26.3	152	n/a	n/a	n/a
N5	Natto	soy	16.5	11.1	12.5	2.5	n/a	n/a	n/a
Meat									
BM1	Beef mince 1	beef	19.9	17	0	71	n/a	n/a	n/a
BM2	Beef mince 2								
BS	Beef steak		19	19	0	58			
CT1	Chicken thigh 1	chicken	27	14	0	82	n/a	n/a	n/a
CT2	Chicken thigh 2								
CB	Chicken breast 1								
PL	Pork loin	pork	27	14	0	62	n/a	n/a	n/a
PS	Pork sirloin								
PC	Pork cutlet								

MS = Meat Substitute, T = Tempeh, TO = Tofu, N = Natto, BM = Beef Mince, BS = Beef Steak, CT = Chicken Thigh, CB = Chicken Breast, PL = Pork Loin, PS = Pork Sirloin, PC = pork cutlet, n/a = information not available, 1, 2—different brands of the same product type.

**Table 2:** Sensory descriptors of meat substitutes and traditional plant-based high-protein food products (n = 5).

Product	Type	Brand	Main Ingredient	Aroma	Flavor/Taste	Texture	Appearance	Mouthfeel
MS1	burger	cooked	black beans	beany, slight smoky, grainy, fresh vegetables, not much aroma	spicy, pleasant, not like beef, salty, beany, lentils, sweet, grainy, beetroot	firm, chewy, hard particles, stay intact upon cutting, soft, crumbly particles, grainy particles, firm on plate, falls in pieces in mouth	red/purple and black particles, nice browning, visible grains, dark, pink, layered pieces (beans pieces)	not fatty, dry, not juicy
MS2	burger	cooked	vegetables	oregano, strong rosemary, herbs, not meat-like, spicy, beany, strong, curry, mustard	salty, sweet, herbs, like stuffing, not unpleasant, starchy, MSG, sweet, taste like mix of vegetables	soft, chewy, cohesive, sticky, stay intact upon cutting, soft, oily, residue in mouth, tooth packing	yellow/orange/brown/grainy, visible green and carrot pieces, veg chunks like in veg burgers	soft, oily
MS3	burger	cooked	brown rice	not meat-like, brown rice smell, guaiacol, cooked grains, spicy, smoky, beany, rosemary, cooked vegetables, very grainy, spices (pepper), seeds	salty, rice flavor, very strong grainy taste, acidic, cereal, aftertaste not beefy, very salty, not great flavor, starchy, herbal, peppery, rye	medium firm, chewy, stay intact upon cutting, soft, grainy particles	visible rice grains, grainy, brown color, dark, flaky	crumbly
MS4	burger	cooked	soy protein	herbal, mild beefy, spicy, overpowering, mushroom	bland, spicy, starchy, beany aftertaste, parsley, spicy paprika—not really that nice, herbal, spices, a bit salty, grainy, very spicy	compact, cohesive, chewy, soft, sticky, dense, soft, not too dry, no particles—processed, first bite is nice then too much processing needed	red color, looks dry, thick and round edges, nice browning, red beef color, like burger, thick dense raw meat appearance	not fatty, not juicy, sucks saliva
MS5	burger	cooked	pea protein	meaty, slight-strong smoky, tomato, onion, beefy, not pleasant, strong off, acidic, artificial, smells like meat	slightly salty, umami, meaty, mushroom, spicy, off, strong aftertaste, smoky, cat food, grainy, aftertaste	soft, meat-like texture, looks good, looks like meat, texture like a burger, tender	homogenous but can see particles, good browning, good brown color, meaty appearance	juicy, oily, not dry
MS6	mince	cooked	mycoprotein	strong acidic, grainy, acetic acid, mild mushroom, starchy, cardboard, strong grainy, wet paper	not nice taste, very bland, strong acidic, weird taste, not meaty, cardboard, cereals, some bitterness, not salty, acidic	powdery, sticky, firm bite, moist, resistant to chew, chewy, crumbly, soft, very small particles	looks like mince, good brown color, meat color	dry, crumbly, not too dry
MS7	mince	cooked	soy protein	vomit, cooked grain smell, not meat like, off, oily, starchy, revolting aroma, cardboard	salty, spicy, off, vomit, chemical/artificial, very bread-like aftertaste	good texture, springy pieces, holds tongue well, chewy, pasty, bouncy	looks like mince, brown	soft
MS8	sausage	cooked	wheat, gluten and soy	herbs, guaiacol, cloves, a bit grainy, starchy, spices, overpowering, peppery	salty, sweet, starchy, spicy, bland, spicy aftertaste	firm first bite, then teeth sinking, soft inside, grainy, dissolving, chewy particles before swallow, soft, rubbery, very compact, missing crispiness on the outside	like sausage, highly processed, pasty, meaty appearance, sticky, looks like a plastic sausage	oily but not juicy, dry, pasty, mouthcoating, not fatty
MS9	sausage	cooked	soy	strong aniseed, herbs, fennel, oily, spices, very aromatic	strong flavor, artificial clove flavor (disgusting), very herby taste, overpowering, herbs, spices, starchy, too herby, acidic, too strong, salty, fennel flavor	oily and firm, homogenous first bite, first bite is like biting into a real sausage, juicy and soft	looks like sausage, chargrilled outside, color contrast inside, looks meaty, good burning on surface	soft, moist not juicy mouthcoating, juicy
MS10	roast (pork)	cooked	wheat, gluten and soy	meaty, herbs, pleasant fried smell, starchy, oily, grainy, acidic, artificial	salty, sweet, taste starchy, acidic, very sweet, not natural, not like meat, very acidic, extremely sweet	a bit firm on the first bite then soft, chewy, good meaty texture, pork texture, meaty texture, very fibrous, good meat like texture, no residue	light brown color, fibrous, not meat-like, pale, very pale	soft, rubbery, quite gummy, a bit moist
T1	tempeh	cooked	chickpea	fermented, very strong cider notes, grilled, baked, acidic, slightly beany	sour, umami, very acidic	crumbly paste, fine particles, soft	golden light	starchy, small particles
T2	tempeh	cooked	fava beans	grilled meat, tortilla, slightly beany, flour, yeasty, fermented	acidic, nutty, umami, mild meaty, beany	dry powder, soft	golden light	slightly crumbly, starchy, small particles
T3	tempeh	cooked	organic split pea and brown rice	slight grilled, meaty, fermented	sauerkraut, slightly bitter, meat-like, umami	fine particles, soft	golden, light	small particles
T4	tempeh	cooked	soy	cereal like, mild baked notes, mild grilled, mild fermented, mild aroma	slightly acidic, nutty, starchy, beany	crumbly, soft	light golden	paste, small particles, slightly chewy, dry
TO1	tofu	cooked	soy	beany, baked notes, mild fried fat	bitter, beany, soy taste, bland, eggy	firm, springy	golden, fried	springy, firm
TO2	tofu silken	cooked	soy	beany, cooked bread, mild aroma, slightly nutty	soy like, bland, mild beany, green, slightly nutty	soft	pale brown, golden, fried	soft, silky, moist
N1	natto	raw	soy	fruity, caramel, banana, green, cheesy, esters	bitter, beany, salty	soft, slimy, beany	brown, stringy, soy seeds	gooey, slightly slimy, soft
N2	natto	raw	soy	ammonia, caramel, coffee	bitter, beany, vomit, acidic, coffee	slimy, sticky, beany	brown, stringy, soy seeds	gooey, slightly slimy, soft
N3	natto	raw	soy	savory, caramel, coffee, BBQ sauce	beany, coffee, bitter, mouth irritating, plain,	soft, slimy, sticky	brown, stringy, soy seeds	gooey, slightly slimy, soft
N4	natto	raw	soy	chocolate, caramel, acidic, mild off	chocolate, coffee, beany, very bitter	slimy, sticky, beany	brown, stringy, soy seeds	gooey, slightly slimy, soft
N5	natto	raw	soy	strong caramel, coffee, cheesy, fermented	very bitter, beany, cassoulet taste (meaty dish)	slimy, sticky, beany	brown, stringy, soy seeds	gooey, slightly slimy, soft
N1	natto	cooked	soy	coffee, caramel	coffee, bitter, sweet, nutty	slimy, sticky, beany	brown, stringy, soy seeds	gooey, slightly slimy, soft
N2	natto	cooked	soy	savory, coffee, oily	sweet, chocolate, slightly bitter, chocolate, savory	slimy, sticky, beany	brown, stringy, soy seeds	gooey, slightly slimy, soft
N3	natto	cooked	soy	meaty, mushroom, smoked ham, tempeh smell	savory, moderate bitter, bacon, mild coffee, meaty, nutty	slimy, sticky, beany	brown, stringy, soy seeds	gooey, slightly slimy, soft
N4	natto	cooked	soy	meaty, caramel, fermented	savory, soy sauce, moderate bitter	slimy, sticky, beany	brown, stringy, soy seeds	gooey, slightly slimy, soft
N5	natto	cooked	soy	coffee	strong coffee taste, sweet, bitter	slimy, sticky, beany	brown, stringy, soy seeds	gooey, slightly slimy, soft

MS = Meat Substitute, T = Tempeh, TO = Tofu, N = Natto, BM = Beef Mince, BS = Beef Steak, CT = Chicken Thigh, CB = Chicken Breast, PL = Pork Loin, PS = Pork Sirloin, PC = pork cutlet.

## Supplementary Materials

The following are available online at https://www.mdpi.com/article/10.3390/foods10040801/s1, Table S1: Statistical analysis of the volatile metabolites measured by SPME GC-MS, Table S2: Statistical analysis of the non-volatile metabolites measured by LC-MS.
