# Supplementary material for: Flavor and Metabolite Profiles of Meat, Meat Substitutes, and Traditional Plant-Based High-Protein Food Products Available in Australia
Source: Foods. 2021 Apr 8;10(4):801. doi: 10.3390/foods10040801 (PMC8068397; doi:10.3390/foods10040801)
Supplement: Supplementary file 1 [file foods-10-00801-s001.pdf]

Supplementary data

Table S1. Statistical analysis of the volatile metabolites measured by SPME GC-MS.

Group	Compound	m/z	LRI	Beef	Chicken	Pork	Substitutes	Natto	Natto cooked	Tempe cooked	Tofu cooked	p-value	LSD
acids	acetic acid	45	1432	98.00	52.00	104.00	394.00	816.00	772.00	472.00	5690.00	0.015	2581.600
	butanoic acid	60	1520	12.88	7.74	20.94	12.14	8.72	9.88	36.98	27.16	0.012	18.572
	isovaleric acid	60	1534	2.00	0.00	0.00	20.00	1070.00	1078.00	38.00	246.00	< 0.001	364.400
	hexanoic acid	60	1855	64.00	26.00	34.00	96.00	18.00	10.00	396.00	432.00	0.017	290.000
	octanoic acid	60	2069	14.00	26.00	22.00	98.00	22.00	10.00	438.00	74.00	0.027	282.000
	nonanoic acid	60	2166	14.00	20.00	20.00	44.00	12.00	18.00	376.00	82.00	0.013	228.800
	Total acids	NA	NA	204.88	131.74	200.94	664.14	1946.72	1897.88	1756.98	6551.16		NA
alcohols	ethanol	45	943	10.00	90.00	2.00	504.00	204.00	102.00	27788.00	1786.00	< 0.001	6000.800
	1-butanol	56	1174	0.50	2.70	1.64	3.38	0.16	0.00	44.64	0.00	< 0.001	10.960
	1-pentanol	55	1290	93.40	44.20	22.20	172.00	3.00	9.00	153.00	164.40	0.003	135.800
	1-hexanol	56	1371	106.00	178.00	106.00	728.00	134.00	122.00	456.00	5600.00	< 0.001	1926.400
	3-octanol	83	1399	0.00	0.00	0.00	7.60	42.80	34.20	3.00	375.80	0.002	149.600
	1-octen-3-ol	57	1431	144.00	430.00	142.00	980.00	718.00	686.00	356.00	8090.00	0.007	3429.200
	1-heptanol	70	1435	31.20	39.60	30.60	65.20	13.80	17.20	104.80	351.40	0.005	144.360
	2,3-dimethyl-5-ethylpyrazine	135	1438	11.00	10.20	16.00	9.80	87.80	163.40	133.60	12.80	< 0.001	70.920
	1-octanol	69	1482	14.60	31.80	8.20	29.80	16.20	22.00	91.60	88.60	0.003	48.800
	(E)-2-octen-1-ol	57	1513	5.00	18.20	6.40	9.60	25.20	14.80	23.20	82.20	0.004	32.240
	2-furanmethanol	98	1530	0.40	1.40	0.00	53.40	54.80	48.20	13.60	26.00	0.058	56.000
	nonanol	51	1539	166.00	88.00	80.00	120.00	48.00	8.00	182.00	620.00	0.005	250.400
	2-phenylethylacohol	91	1916	4.00	8.00	2.00	42.00	94.00	88.00	3218.00	164.00	< 0.001	975.200
	Total alcohols	NA	NA	586.10	942.10	417.04	2724.78	1441.76	1314.80	32567.44	17361.20		NA
aldehydes	acetaldehyde	44	702	44.00	64.00	70.00	52.00	326.00	328.00	1364.00	62.00	< 0.001	222.400
	2-methylpropanal	72	812	31.00	23.40	16.00	19.20	0.00	33.00	60.80	0.00	< 0.001	28.840
	2-methylbutanal	41	912	156.80	123.60	93.40	111.20	19.60	167.20	305.40	44.20	0.003	143.680
	3-methylbutanal	58	917	69.20	50.00	49.80	132.80	16.20	154.20	221.40	21.80	< 0.001	110.800
	pentanal	44	981	62.80	201.40	59.80	135.20	0.00	0.00	233.80	162.40	0.021	173.960
	2-butenal	70	1045	2.00	0.00	2.00	14.00	2.00	6.00	5968.00	30.00	< 0.001	1518.000
	hexanal	56	1091	458.00	2008.00	688.00	1114.00	16.00	30.00	1002.00	1518.00	0.002	1071.600

Group	Compound	m/z	LRI	Beef	Chicken	Pork	Substitutes	Natto	Natto cooked	Tempe cooked	Tofu cooked	p-value	LSD
	2-methyl-2-butenal	84	1106	0.20	0.00	0.20	29.40	0.00	1.20	182.40	0.00	< 0.001	60.960
	heptanal	70	1196	59.40	123.60	61.60	169.40	1.60	9.60	197.20	182.80	0.009	152.880
	octanal	56	1305	42.80	74.80	42.40	79.60	2.80	15.20	158.80	75.60	0.003	82.320
	(E)-2-heptenal	56	1346	35.40	22.20	53.20	23.60	7.00	14.80	209.00	250.40	< 0.001	99.800
	nonanal	57	1402	150.00	324.00	244.00	238.00	30.00	58.00	428.00	326.00	0.116	326.800
	(E,E)-2,4-hexadienal	81	1408	6.00	24.00	10.00	114.00	1358.00	1894.00	356.00	38.00	< 0.001	848.800
	furfural	95	1439	8.60	6.20	2.80	186.00	8.40	13.00	57.00	33.20	0.016	170.960
	benzaldehyde	106	1466	176.00	130.00	122.00	322.00	310.00	2946.00	388.00	246.00	< 0.001	635.200
	(E)-2-nonenal	83	1474	1.00	0.40	0.00	20.80	0.80	1.00	54.80	48.20	< 0.001	31.640
	(E,E)-2,4-decadienal	81	1599	2.00	6.00	0.00	84.00	0.00	72.00	1946.00	798.00	0.005	1173.600
	Total aldehydes	NA	NA	1261.20	3117.60	1445.20	2793.20	1772.40	5415.20	11768.60	3774.60		NA
diols	2,3-butanediol	45	1364	0.00	0.00	0.00	4.00	8.00	8.00	988.00	0.00	< 0.001	521.200
	1,3-butanediol	45	1493	10.00	2.00	2.00	112.00	84.00	126.00	1156.00	98.00	< 0.001	438.400
	Total diols	NA	NA	10.00	2.00	2.00	116.00	92.00	134.00	2144.00	98.00		NA
esters	ethyl acetate	43	888	0.00	2.00	0.00	28.00	2.00	0.00	1614.00	192.00	< 0.001	750.000
	propanoic acid, ethyl ester (ethyl propionate)	43	969	1.00	2.80	3.40	12.40	6.00	4.20	157.20	12.60	< 0.001	43.800
	methyl butanoate	74	993	27.80	15.00	16.40	41.60	10.00	17.00	25.60	115.80	0.005	45.920
	Butanoic acid, 2-methylethyl ester (ethyl-2-methyl butanoate)	57	1062	0.00	0.00	0.00	2.00	12.00	12.00	718.00	0.00	< 0.001	219.200
	Butanoic acid, 3-methylethyl ester ethyl-3-methyl butanoate)	88	1079	0.00	0.00	0.00	0.40	4.60	4.60	319.20	0.00	< 0.001	162.680
	Hexanoic acid, ethyl ester (ethyl hexanoate)	88	1247	0.00	5.20	1.00	4.80	0.00	0.00	115.40	0.00	< 0.001	42.760
	Heptanoic acid, ethyl ester (ethyl heptanoate)	88	1347	1.80	0.00	0.00	0.20	183.80	28.40	23.00	0.00	0.009	124.840
	octyl acetate (octyl ethanoate)	70	1387	13.00	89.40	80.20	4.40	4.00	8.40	221.40	61.60	< 0.001	72.960
	octanoic acid, ethyl ester (ethyl octanoate)	88	1419	0.00	0.00	0.00	1.60	0.00	0.00	236.40	0.00	< 0.001	67.280
	benzoic acid, methyl ester (methyl benzoate)	105	1513	0.00	0.00	0.00	4.40	31.00	6.80	275.80	23.20	< 0.001	35.320
	Total esters	NA	NA	43.60	114.40	101.00	99.80	253.40	81.40	3706.00	405.20		NA
furans	2-methylfuran	82	870	3.40	0.00	0.00	89.00	0.00	0.00	0.60	24.80	0.028	91.840
	2-ethylfuran	81	958	18.00	6.00	0.00	434.00	40.00	16.00	68.00	1190.00	< 0.001	489.200
	2-butylfuran	81	1137	3.20	3.00	0.00	90.80	2.60	0.00	27.00	205.80	< 0.001	89.640

Group	Compound	m/z	LRI	Beef	Chicken	Pork	Substitutes	Natto	Natto cooked	Tempe cooked	Tofu cooked	p-value	LSD
	2-pentylfuran	81	1243	78.00	60.00	46.00	2388.00	10.00	26.00	1366.00	7902.00	< 0.001	3104.800
	acetylfuran	95	1461	4.80	12.80	6.60	60.60	0.00	6.60	13.80	0.00	< 0.001	26.280
	Total furans	NA	NA	107.40	81.80	52.60	3062.40	52.60	48.60	1475.40	9322.60		NA
ketones	acetone	43	816	128.00	122.00	100.00	238.00	1166.00	432.00	218.00	236.00	< 0.001	295.600
	2-butanone	43	903	104.20	105.80	105.60	143.00	120.00	82.00	421.20	135.00	< 0.001	116.680
	2,3-butanedione	43	981	80.00	88.00	64.00	174.00	5004.00	5516.00	600.00	132.00	< 0.001	1859.200
	2,3-pentanedione	57	1074	33.60	7.80	7.80	27.60	8.20	22.60	52.80	0.00	0.103	37.680
	2-heptanone	43	1187	36.00	36.00	30.00	338.00	352.00	306.00	82.00	258.00	< 0.001	239.600
	6-methyl-2-heptanone	43	1250	5.80	11.40	4.20	9.60	358.80	296.80	5.20	24.60	< 0.001	60.440
	3-octanone	99	1281	0.00	0.00	0.00	13.20	9.60	13.60	16.20	101.20	0.022	47.280
	2-octanone	58	1313	17.20	17.60	19.20	98.00	18.80	16.60	36.80	74.00	< 0.001	55.480
	3-hydroxy-2-butanone (acetoin)	45	1323	174.00	0.00	12.00	116.00	4852.00	4954.00	2126.00	0.00	< 0.001	3177.200
	1_octen_3_one ???	55	1327	0.40	6.80	2.40	12.20	2.80	2.60	49.60	53.60	< 0.001	28.760
	2-acetyl - 1-pyrroline	83	1357	0.32	0.18	3.12	6.48	4.24	0.18	13.76	0.00	0.364	13.428
	(E,E)-3,5-octadien-2-one	95	1467	0.00	0.00	0.00	608.00	4.00	10.00	38.00	0.00	0.099	730.000
	Total ketones	NA	NA	579.52	395.58	348.32	1784.08	11900.44	11652.38	3659.56	1014.40		NA
phenols	guaiacol	109	1859	2.00	14.00	8.00	50.00	752.00	496.00	0.00	0.00	< 0.001	224.800
	p-cresol	107	2090	43.80	53.80	39.80	86.00	59.40	78.80	187.20	156.80	0.01	89.360
	Total phenols	NA	NA	45.80	67.80	47.80	136.00	811.40	574.80	187.20	156.80		NA
pyrazine	methylpyrazine	94	1306	18.20	2.80	17.00	45.20	18.00	16.40	19.40	2.40	0.002	27.480
	2,5-dimethylpyrazine	108	1351	50.00	30.00	74.00	40.00	17950.00	16502.00	30.00	56.00	< 0.001	2705.200
	2,6-dimethylpyrazine	108	1352	24.00	10.00	36.00	50.00	0.00	0.00	38.00	56.00	0.532	2349.200
	2-ethyl-5-methylpyrazine	121	1402	25.00	14.20	33.20	18.40	119.00	113.00	21.60	26.60	< 0.001	32.520
	trimethylpyrazine	122	1415	46.00	34.00	74.00	66.00	10478.00	9128.00	112.00	2.00	< 0.001	2734.000
	3-ethyl-2-5-dimethylpyrazine	135	1429	38.40	34.20	58.60	24.80	641.40	613.40	52.80	12.80	< 0.001	121.120
	tetramethylpyrazine	136	1446	0.00	0.00	0.00	6.00	9176.00	9076.00	38.00	0.00	< 0.001	4710.800
	2,3,5-trimethyl-6-ethylpyrazine	149	1464	0.00	2.00	2.00	2.00	430.00	556.00	42.00	0.00	< 0.001	210.800
	methoxypyrazine	110	1493	3.00	3.60	1.60	46.80	0.00	0.20	1.40	0.00	< 0.001	35.360
	Total pyrazine	NA	NA	201.60	127.20	294.80	252.40	38812.40	36004.80	353.80	155.80		NA
sulfur	methanethiol	48	691	11.06	40.84	42.02	2.62	3.92	4.80	6.92	4.58	< 0.001	13.172
	carbon disulphide	76	726	26.40	79.40	143.80	32.80	46.40	47.80	12.20	88.60	0.033	77.920
	dimethylsufide	62	747	4.20	2.00	0.20	4.80	0.00	0.00	0.00	55.20	0.003	23.080
	dimethyl- disulfide	94	1085	9.40	3.00	7.40	66.80	11.00	14.20	13.40	0.00	< 0.001	33.880

Group	Compound	m/z	LRI	Beef	Chicken	Pork	Substitutes	Natto	Natto cooked	Tempe cooked	Tofu cooked	p-value	LSD
other	dimethyl trisulphide	126	1393	3.60	0.00	1.40	11.40	12.80	10.00	27.40	19.20	0.147	20.480
	methanethiol	48	1435	7.66	6.58	4.06	5.30	0.28	18.08	18.90	0.00	< 0.001	10.140
	Total sulfur	NA	NA	62.32	131.82	198.88	123.72	74.40	94.88	78.82	167.58		NA
	octane	43	794	108.00	64.00	200.00	334.00	4.00	30.00	252.00	462.00	< 0.001	227.600
	2-ethylthiophen	97	1103	1.20	2.60	0.00	7.60	8.60	3.40	0.00	44.60	0.007	19.080
	1,2-dimethyl benzene	91	1137	4.00	11.60	9.60	12.00	13.20	15.40	180.20	36.80	< 0.001	57.840
	UNKNOWN	43	1149	7.00	4.00	4.40	5.40	445.80	325.80	0.00	0.00	< 0.001	89.480
	1,3-dimethylbenzene	91	1184	0.00	8.20	6.00	14.80	11.80	14.40	169.20	2.40	< 0.001	47.720
	d-limonene	68	1200	12.00	0.00	0.00	2042.00	0.00	0.00	0.00	0.00	0.021	2090.400
	pyridine	79	1211	23.80	42.40	38.00	123.60	10.80	9.00	29.40	83.40	0.119	128.080
	UNKNOWN	81	1211	14.00	0.00	12.00	386.00	96.00	74.00	36.00	74.00	0.039	373.600
	eucalyptol	79	1219	0.00	0.00	0.00	496.00	0.00	0.00	38.00	0.00	0.003	432.400
	trimethyl oxazole	111	1220	0.00	0.00	0.00	354.00	318.00	292.00	2.00	0.00	0.022	360.400
	caryophyllene	93	1505	0.00	0.00	0.00	194.20	0.00	0.00	106.40	0.00	< 0.001	139.760
	butyrolactone	42	1522	21.20	35.20	29.60	14.20	140.00	117.20	77.40	41.00	< 0.001	84.120
	maltol	126	1959	8.00	0.00	0.00	116.00	560.00	696.00	408.00	670.00	< 0.001	412.400

Table S2. Statistical analysis of the non-volatile metabolites measured by LC-MS.

Compound	MW	RT	Beef	Chicken	Pork	Meat substitutes	Natto	Tempeh	Tofu	p-value	LSD	Notes
Stachydrine	143.09446	6.655	0	189	46	152	55	30	0	0.047	153.8	
Phenylalanine	165.07868	6.967	491	450	400	547	0	0	118	< 0.001	382.6	bitter, sweet [1,2]
Tryptophan*	204.08956	7.064	38	69	38	449	1038	2057	70	< 0.001	788.8	bitter, sweet [2]
Leucine*	131.09448	7.165	1059	1123	799	1933	8413	5772	68	< 0.001	2064.2	bitter leucine [1]
Isoleucine	131.09446	7.291	560	776	453	967	6509	5336	62	< 0.001	1692.8	
Methionine*	149.05085	7.382	251	457	296	280	1976	763	14	< 0.001	558.0	sweet [1]
Acetyl-L-carnitine	203.11536	7.399	22156	3361	3026	34	1	417	2	< 0.001	1996.4	
Tyrosine*	181.07364	7.447	15	35	49	71	389	41	5	< 0.001	155.8	bitter, umami [1]
Proline*	115.06328	7.528	966	2529	1148	4021	1267	11366	208	< 0.001	2631.8	sweet/neutral
Valine	117.07874	7.715	0	0	0	0	0	118	0	0.238	110.2	bitter [1,2]
Glutamic acid	147.05280	8.020	405	1240	460	1921	1515	2220	281	0.402	2261.2	kokumi/umami [1,3]
Aspartic acid*	133.03733	8.038	5	37	18	37	62	136	3	< 0.001	32.0	umami [3]
ALANINE	89.04784	8.122	319	536	334	977	753	3118	84	< 0.001	814.2	sweet [2]
Homoserine	119.05818	8.129	163	463	171	91	1060	1677	19	< 0.001	360.8	
Carnitine	161.10479	8.142	38833	7071	15747	390	39	3714	77	< 0.001	2067.0	
Creatine	131.06913	8.254	20817	22105	22364	45	0	47	0	< 0.001	1184.8	
Glycine*	75.03224	8.339	25	46	24	7	75	66	0	< 0.001	28.2	sweet [1,2]
Glutamine	146.06902	8.366	1375	761	674	896	159	2655	65	0.019	1485.4	kokumi/umami [1]
Serine*	105.04263	8.414	98	144	76	66	310	311	3	< 0.001	105.0	sweet [2]
Asparagine*	132.05332	8.435	28	34	21	172	359	358	9	< 0.001	127.4	
Citrulline	175.09540	8.547	27	25	64	76	7	379	20	< 0.001	89.2	
Arginine	174.11136	8.765	0	0	0	2976	124	1977	2385	< 0.001	2192.2	
Acetylarginine	216.12193	8.896	0	4	0	12	147	123	2	< 0.001	36.2	
Ƴ-Aminobutyric acid (GABA)	103.06357	9.232	47	2048	36	1640	84	6265	349	< 0.001	1635.2	
4-Guanidinobutyric acid	145.08488	9.442	2	222	0	199	190	156	44	0.002	142.8	
Histidine*	155.06925	12.884	129	247	93	175	2597	2203	45	< 0.001	483.4	sweet [2]
Lysine*	146.10528	13.479	366	600	311	385	2917	4057	32	< 0.001	748.6	bitter [1]
Ornithine*	132.08977	13.578	423	61	63	369	2047	2468	21	< 0.001	775.2	
L-Pyroglutamic-acid	129.04247	8.015	0	63	17	77	128	98	0	0.031	90.8	
N-N-Dimethylglycine	103.06337	8.120	0	28	0	30	39	69	24	0.032	41.6	

Compound	MW	RT	Beef	Chicken	Pork	Meat substitutes	Natto	Tempeh	Tofu	p-value	LSD	Notes
Val-Asp	232.10561	4.410	0	0	0	0	141	0	0	< 0.001	32.6	dipeptidyl peptidase IV inhibitor (DPP IV inhibitor) [4]
Pro-Thr	216.11067	7.097	0	0	0	0	14	14	0	< 0.001	8.6	dipeptidyl peptidase IV inhibitor (DPP IV inhibitor) [4]
Phe-Tyr	328.14165	7.116	0	0	0	1	14	3	0	< 0.001	3.4	ACE inhibitor [4]
Leu-Leu	244.17824	7.186	0	2	10	141	1857	238	0	< 0.001	429.0	dipeptidyl peptidase IV inhibitor (DPP IV inhibitor) [4]
Tyr-leu	294.15743	7.296	0	0	0	10	63	12	0	< 0.001	21.0	
Leu-Pro	228.14692	7.304	125	0	130	44	27	241	0	0.038	162.6	
Pro-Leu	228.14688	7.445	159	399	391	2	127	0	0	< 0.001	101.6	
Ile-His	268.15304	7.524	0	0	0	4	186	89	0	< 0.001	71.2	DPP-III inhibitor [4]
Val-Met	248.11905	7.693	0	0	0	2	52	5	0	< 0.001	15.4	dipeptidyl peptidase IV inhibitor (DPP IV inhibitor) [4]
Val-Pro	214.13141	7.778	4	0	19	3	18	0	0	< 0.001	12.4	dipeptidyl peptidase IV inhibitor (DPP IV inhibitor) , ACE inhibitor [4]
Val-Val	216.14708	7.794	37	7	53	56	471	133	0	< 0.001	141.4	
Oglufanide	333.13185	7.799	0	0	0	0	9	0	0	< 0.001	2.0	
Pro-Met	246.10361	7.838	8	4	12	9	158	6	0	< 0.001	54.8	dipeptidyl peptidase IV inhibitor (DPP IV inhibitor) [4]
γ-Glu-Leu	260.13684	7.882	0	1	11	10	236	26	0	< 0.001	58.0	kokumi
ϒ-Glu-Cys	250.06165	7.911	0	8	4	0	0	0	0	< 0.001	1.4	kokumi
Val-Pro	214.13143	7.973	27	86	28	156	316	93	0	0.023	204.0	
ϒ-Glu-Glu	276.09524	8.084	0	2	0	7	6	20	0	< 0.001	8.2	kokumi
Gly-Phe	222.10012	8.120	0	0	0	3	89	32	0	< 0.001	19.2	bitter; dipeptidyl peptidase IV inhibitor (DPP IV inhibitor) [4]
Leu-Glu	260.13673	8.140	3	6	21	38	148	442	0	< 0.001	80.2	bitter [4]
Ala-Tyr	252.11057	8.144	0	0	2	7	34	21	0	< 0.001	10.4	dipeptidyl peptidase IV inhibitor (DPP IV inhibitor) [4]
Pro-Leu	228.14684	8.160	98	297	315	27	13	11	11	< 0.001	40.0	
Thr-Tyr	282.12113	8.220	0	0	0	1	17	4	0	< 0.001	3.0	dipeptidyl peptidase IV inhibitor (DPP IV inhibitor) [4]
Lys-Leu	259.18917	8.343	0	0	0	11	225	3	3	< 0.001	69.4	anti-diabetic agent [5]
Val-Glu	246.12109	8.354	6	0	6	7	49	66	0	< 0.001	26.4	bitter; dipeptidyl peptidase IV inhibitor (DPP IV inhibitor) [4]
Gln-Trp	332.14782	8.408	0	0	0	0	10	0	0	< 0.001	2.2	dipeptidyl peptidase IV inhibitor (DPP IV inhibitor) [4]
Glu-Pro	244.10555	8.441	0	3	0	43	179	110	0	< 0.001	88.8	DPP IV inhibitor Tamam et al [4,5]

Compound	MW	RT	Beef	Chicken	Pork	Meat substitutes	Natto	Tempeh	Tofu	p-value	LSD	Notes
Ala-Pro	186.10016	8.593	32	44	35	58	552	43	0	< 0.001	198.4	dipeptidyl peptidase IV inhibitor (DPP IV inhibitor) [4]
Gln-Tyr	309.13188	8.611	0	0	0	0	33	2	0	< 0.001	8.8	dipeptidyl peptidase IV inhibitor (DPP IV inhibitor) [4]
Thr-Pro	216.11066	8.620	4	7	4	10	124	10	0	< 0.001	43.4	ACE inhibitor [4]
Leu-Gln	259.15279	8.647	0	1	6	3	411	57	0	< 0.001	81.6	ACE inhibitor [4]
Ser-Glu	234.08485	8.749	1	0	1	0	30	13	0	< 0.001	6.6	
Val-Glu	245.13716	8.841	9	3	8	5	154	41	0	< 0.001	36.8	
Val-Asn	231.12155	8.869	0	0	0	0	15	4	0	< 0.001	7.0	dipeptidyl peptidase IV inhibitor (DPP IV inhibitor) [4]
Pro-Gln	243.12153	8.874	3	3	3	2	447	38	0	< 0.001	149.8	dipeptidyl peptidase IV inhibitor (DPP IV inhibitor) [4]
Ala-Thr	190.09507	8.886	14	2	13	8	178	54	0	< 0.001	52.4	
Ala-Glu	217.10579	9.107	33	163	97	3	239	58	0	< 0.001	58.4	
Val-Lys	245.17362	9.891	0	0	0	0	435	8	11	< 0.001	126.0	
Pro-Pro	212.11632	10.687	0	0	0	31	0	0	0	0.328	48.6	bitter; Alpha-glucosidase inhibitor [4]
Arg-Ala	245.14852	12.115	0	0	0	0	170	0	0	< 0.001	52.4	salt enhancer ; ACE inhibitor, activating ubiquitin-mediated proteolysis, dipeptidyl peptidase IV inhibitor (DPP IV inhibitor) [4]
Val-Arg	273.17940	13.492	5	1	0	0	0	0	0	0.004	2.8	salt enhancer; dipeptidyl peptidase IV inhibitor (DPP IV inhibitor) [4]
Carnosine	226.10602	13.739	4829	2872	6273	0	0	0	0	< 0.001	670.4	
Arg-Val	273.17966	13.751	0	0	0	0	291	0	0	< 0.001	86.0	salt enhancer; DPP-III inhibitor [4]
Gln-His	283.12768	13.968	0	0	0	0	47	9	0	< 0.001	18.2	dipeptidyl peptidase IV inhibitor (DPP IV inhibitor) [4]
Gly-Lys	203.12682	14.395	0	0	0	0	97	9	0	< 0.001	28.0	dipeptidyl peptidase IV inhibitor (DPP IV inhibitor) [4]
Val-His	254.13746	14.761	17	4	17	4	95	22	0	< 0.001	27.0	DPP IV inhibitor [4,5]
Glycitein	284.06798	1.475	0	0	0	24	96	1	232	< 0.001	38.4	
Chrysin	254.05746	1.494	0	0	0	73	240	97	465	< 0.001	128.4	
Apigenin	270.05232	1.596	0	0	0	0	3	3	27	< 0.001	3.6	
Genistin	432.10486	2.119	0	0	0	11	77	11	68	< 0.001	19.0	
Daidzin	416.10993	2.362	0	0	0	173	367	26	312	< 0.001	199.4	
2-Deoxyadenosine	251.10145	2.695	0	0	0	175	606	385	0	< 0.001	177.0	
Adenosine*	267.09628	3.047	0	11	0	1089	2781	1370	138	< 0.001	703.4	
Adenine*	135.05428	3.064	10	37	1	2699	823	3010	175	< 0.001	1469.8	
7-Methylguanine	136.03821	3.180	0	0	0	39	163	44	0	< 0.001	35.2	

Compound	MW	RT	Beef	Chicken	Pork	Meat substitutes	Natto	Tempeh	Tofu	p-value	LSD	Notes
Hypoxanthine*	165.06491	3.180	7991	6236	3498	244	241	1059	149	< 0.001	1945.8	
Inosine*	268.08010	4.338	638	669	1199	80	198	112	0	< 0.001	285.2	
Guanine	151.04919	4.715	0	134	41	240	523	19	27	< 0.001	214.2	
Guanosine*	283.09116	6.061	0	33	61	386	591	158	0	< 0.001	250.4	
Cytosine	111.04323	6.440	23	15	43	596	67	48	0	0.006	516.4	
Cytydine*	243.08504	6.440	19	10	34	671	57	34	0	0.011	621.6	
Inosine-5-monophosphate (IMP)*	348.04603	7.824	41	101	149	1	0	0	0	< 0.001	34.4	kokumi, umami [3]
Val-Pla-Pro-Gly	342.18972	8.751	0	0	0	0	39	0	0	< 0.001	16.2	
Ala-Ala-Pro-Ala	328.17407	8.973	0	0	0	0	31	0	0	< 0.001	8.0	
Ala-Gly-Ala-Gly	274.12716	9.171	0	0	0	0	282	88	0	< 0.001	47.6	
Ala-Gly-Gly-Gly	260.11159	9.193	0	0	0	0	22	9	0	< 0.001	5.0	
Leu-Ala-Ser-Lys	417.25678	14.693	5	5	5	1	0	0	0	< 0.001	1.6	
Val-Val-Glu	345.18948	2.620	0	0	0	0	105	0	0	< 0.001	45.0	
Gly-Val-Pro	271.15280	2.935	0	0	0	0	3	67	0	< 0.001	15.0	
Pro-Val-Gly	271.15278	3.303	0	0	0	0	0	8	0	< 0.001	3.2	
Ala-Thr-Glu	319.13744	3.306	0	0	0	0	33	0	0	< 0.001	5.4	
Pro-Thr-Ile	329.19444	3.697	0	0	0	0	26	0	0	< 0.001	15.2	
Val-Pro-Leu	327.21514	7.448	0	0	0	3	95	0	0	< 0.001	51.2	dipeptidyl peptidase IV inhibitor (DPP-IV inhibitor), antiamnestic, Stimulating vasoactive substance release [4]
Pro-Val-Val	313.19953	7.642	0	0	0	2	55	0	0	< 0.001	18.2	
Val-Val-Val	315.21524	7.663	0	0	0	0	51	0	0	< 0.001	12.2	bitter; Dvl protein binding [4]
Thr-Pro-Leu	329.19448	7.994	0	0	0	0	43	1	0	< 0.001	7.8	
Glutathione	307.08285	8.009	286	371	311	1	0	0	0	< 0.001	52.4	kokumi [6]
Ala-Pro-Leu	299.18401	8.016	0	0	0	0	190	0	0	< 0.001	61.8	
γ-glu-abu-gly)ophthalmic acid	289.12673	8.023	14	11	5	11	2	6	2	0.068	9.8	kokumi
Thr-Leu-Ile	345.22570	8.024	0	0	0	0	47	0	0	< 0.001	9.4	
Pro-Ser-Phe	349.16317	8.054	0	0	0	0	39	0	0	< 0.001	9.4	
Val-Ala-Trp	374.19470	8.138	0	0	0	0	21	0	0	< 0.001	3.0	
Gly-Leu-Pro	285.16841	8.280	0	0	0	2	162	18	0	< 0.001	40.2	ACE inhibitor [4]
Ala-Glu-Leu	331.17373	8.350	0	0	0	0	18	0	0	< 0.001	3.4	ACE inhibitor [4]
Ala-Leu-Gly	259.15280	8.528	0	0	0	1	605	49	0	< 0.001	113.2	
Glu-Ala-Pro	315.14255	8.678	0	0	0	0	46	0	0	< 0.001	10.4	ACE inhibitor [4]

Compound	MW	RT	Beef	Chicken	Pork	Meat substitutes	Natto	Tempeh	Tofu	p-value	LSD	Notes
Ala-Pro-Ala	257.13714	8.890	0	0	0	1	69	8	0	< 0.001	17.0	
Gly-Thr-Pro	273.13194	9.004	0	0	0	0	41	0	0	< 0.001	8.6	
Val-Val-Ile	329.23073	9.086	0	0	0	0	33	0	0	< 0.001	15.6	
Leu-Ala-Lys	330.22591	9.116	0	0	0	0	44	0	0	< 0.001	13.6	
Pro-Thr-Asn	330.15326	9.198	0	0	0	0	40	0	0	< 0.001	15.2	
Gln-Ala-Ser	304.13779	9.386	0	0	0	0	11	0	0	< 0.001	2.4	
Spermidine	145.15769	7.339	261	177	159	88	148	285	23	0.006	143.8	
Valinol	103.09971	8.432	29	9	49	9	0	55	2	< 0.001	9.0	
Tyramine	137.08385	8.539	1165	0	108	39	0	4570	56	< 0.001	2361.2	
Maltol	126.03174	1.652	0	0	0	16	44	6	3	< 0.001	15.2	
Kynurenic acid	189.04228	2.324	0	0	0	4	0	16	0	0.032	10.6	
Nornicotine	148.09991	5.411	0	0	0	0	118	0	0	< 0.001	35.6	
Creatinine	113.05877	5.593	24560	3552	13497	20	0	0	0	< 0.001	3195.0	
Betaine	117.07884	6.940	23876	26131	22409	17073	33676	15379	2480	< 0.001	12988.0	
Pipecolinic acid	129.07870	7.501	6	15	2	1625	65	144	6	0.764	3603.4	
α-Lactose	342.11562	7.861	0	0	0	110	0	97	320	< 0.001	68.2	
Hypotaurine	109.01965	8.145	3	51	30	0	0	1	0	< 0.001	11.4	
Pangamic acid	281.11050	8.238	0	0	0	32	0	2	0	0.002	26.8	
Choline	103.09969	9.043	2049	4153	3934	31703	13662	54124	30665	< 0.001	20980.8	
Salsolinol	179.09418	9.215	0	0	0	44	0	53	0	0.733	104.6	
Tetramethylpyrazine	136.09993	1.669	0	0	0	0	110	0	0	< 0.001	39.2	
2,5-Dimethylpyrazine	108.06881	1.677	0	0	0	0	135	0	0	< 0.001	34.8	

1. Charve, J.; Manganiello, S.; Glabasnia, A. Analysis of Umami Taste Compounds in a Fermented Corn Sauce by Means of Sensory-Guided Fractionation. *Journal of Agricultural and Food Chemistry* **2018**, *66*, 1863-1871, doi:10.1021/acs.jafc.7b05633.
2. Bachmanov, A.A.; Bosak, N.P.; Glendinning, J.I.; Inoue, M.; Li, X.; Manita, S.; McCaughey, S.A.; Murata, Y.; Reed, D.R.; Tordoff, M.G., et al. Genetics of Amino Acid Taste and Appetite. *Adv Nutr* **2016**, *7*, 806s-822s, doi:10.3945/an.115.011270.
3. Zhang, Y.; Venkitasamy, C.; Pan, Z.L.; Wang, W. Recent developments on umami ingredients of edible mushrooms - A review. *Trends in Food Science & Technology* **2013**, *33*, 78-92, doi:https://doi.org/10.1016/j.tifs.2013.08.002.
4. Minkiewicz, P.; Iwaniak, A.; Darewicz, M. BIOPEP-UWM Database of bioactive peptides: Current opportunities *International Journal of Molecular Sciences* **2019**, *20*, 5978.
5. Tamam, B.; Syah, D.; Suhartono, M.T.; Kusuma, W.A.; Tachibana, S.; Lioe, H.N. Proteomic study of bioactive peptides from tempe. *Journal of Bioscience and Bioengineering* **2019**, *128*, 241-248, doi:https://doi.org/10.1016/j.jbiosc.2019.01.019.
6. Kuroda, M.; Kato, Y.; Yamazaki, J.; Kai, Y.; Mizukoshi, T.; Miyano, H.; Eto, Y. Determination and quantification of the kokumi peptide, gamma-glutamyl-valyl-glycine, in commercial soy sauces. *Food Chem* **2013**, *141*, 823-828, doi:https://doi.org/10.1016/j.foodchem.2013.03.070.
